# Optimization of Dry Sliding Wear in Hot-Pressed Al/B_4_C Metal Matrix Composites Using Taguchi Method and ANN

**DOI:** 10.3390/ma17164056

**Published:** 2024-08-15

**Authors:** Sandra Gajević, Slavica Miladinović, Onur Güler, Serdar Özkaya, Blaža Stojanović

**Affiliations:** 1 University of Kragujevac, Faculty of Engineering, Sestre Janjić 6, 34000 Kragujevac, Serbia; slavicam@kg.ac.rs (S.M.); blaza@kg.ac.rs (B.S.); 2 Faculty of Engineering, Metallurgical and Materials Engineering, Karadeniz Technical University, 61080 Trabzon, Turkey; onurguler@ktu.edu.tr (O.G.); sozkaya@ktu.edu.tr (S.Ö.)

**Keywords:** metal matrix composite, B_4_C, Taguchi, ANN

## Abstract

The presented study investigates the effects of weight percentages of boron carbide reinforcement on the wear properties of aluminum alloy composites. Composites were fabricated via ball milling and the hot extrusion process. During the fabrication of composites, B_4_C content was varied (0, 5, and 10 wt.%), as well as milling time (0, 10, and 20 h). Microstructural observations with SEM microscopy showed that with an increase in milling time, the distribution of B_4_C particles is more homogeneous without agglomerates, and that an increase in wt.% of B_4_C results in a more uniform distribution with distinct grain boundaries. Taguchi and ANOVA analyses are applied in order to investigate how parameters like particle content of B_4_C, normal load, and milling time affect the wear properties of AA2024-based composites. The ANOVA results showed that the most influential parameters on wear loss and coefficient of friction were the content of B_4_C with 51.35% and the normal load with 45.54%, respectively. An artificial neural network was applied for the prediction of wear loss and the coefficient of friction. Two separate networks were developed, both having an architecture of 3-10-1 and a tansig activation function. By comparing the predicted values with the experimental data, it was demonstrated that the well-trained feed-forward-back propagation ANN model is a powerful tool for predicting the wear behavior of Al2024-B_4_C composites. The developed models can be used for predicting the properties of Al2024-B_4_C composite powders produced with different reinforcement ratios and milling times.

## 1. Introduction

The application of various metal matrix composites (MMCs) is steadily increasing due to their superior physical, mechanical, and tribological characteristics compared to their base materials. Lightweight metal-based composites with aluminum, magnesium, and zinc bases are utilized in numerous industries because of their low density [1]. Aluminum matrix composites (AMCs) have gained a lot of attention in the last few decades due to the possibility of obtaining materials with different characteristics. Reinforcements are, usually, oxides, carbides, nitrides, and borides, and they can take the form of particles, whiskers, platelets, or fibers [2]. As reinforcements for aluminum composites are widely used ceramics, such as SiC, Al_2_O_3_, B_4_C, MgO, and MoSi_2_ [3,4,5,6].

The excellent combination of high specific strength and good wear resistance is the reason that aluminum composites are one of the most promising materials for future applications in industry in general, especially in automotive, aerospace, and military industries [7]. In recent years, researchers have focused on investigating AMCs for their potential to enhance tribological characteristics while maintaining good ductility.

This study focuses on the AA2024 alloy, which is extensively utilized as a base material in MMCs due to its superior mechanical properties and processing benefits compared to other metal matrices. AA2024 exhibits an excellent combination of high strength, low density, and good machinability, which are critical for aerospace and automotive applications where weight reduction without compromising strength is paramount [8,9,10]. Additionally, the alloy’s high fatigue resistance and fracture toughness make it a robust choice for structural components subjected to cyclic loading conditions. The presence of copper in AA2024 enhances its age-hardening response, resulting in improved mechanical properties through precipitation-strengthening mechanisms [11]. Moreover, AA2024’s compatibility with various reinforcement materials, such as ceramic particles and fibers, allows for the tailored design of composites to meet specific performance requirements. These attributes underscore the significant advantages of AA2024 as a base material in MMCs, paving the way for the development of high-performance composite materials for demanding engineering applications [12]. Among the various reinforcement particles available for enhancing the properties of MMCs, B_4_C stands out due to its exceptional characteristics. B_4_C is renowned for its extreme hardness, ranking just below diamond and cubic boron nitride, which significantly improves the wear resistance and hardness of the composite material [13]. Additionally, B_4_C particles possess a low density, which is crucial for maintaining the lightweight advantage of AA2024 base composites, a critical factor for aerospace and automotive applications where weight savings are paramount [14]. B_4_C also exhibits excellent chemical stability and a high melting point, which contribute to the thermal stability and corrosion resistance of the composite, enhancing its performance in high-temperature and corrosive environments. Furthermore, the high modulus of elasticity of B_4_C reinforces the matrix, leading to improved stiffness and load-bearing capacity of the composite [15,16]. These attributes collectively underscore the superiority of B_4_C as a reinforcement material in AA2024-based MMCs, enabling the development of composites with outstanding mechanical and thermal properties. Therefore, in the present study, AA2024 has been selected as the base material and B_4_C particles as the reinforcement, due to their combined superior mechanical properties, low density, and enhanced wear resistance, which are critical for advanced MMCs.

In order to better understand the connection between selected factors and their effects, optimization methods and software tools are used in different phases, starting from the production of composites (composition, size, type, and shape of reinforcement, selection of production method, influence of certain parameters during the process itself, such as melting temperature, mixing speed, mixing time, pressure, and many others) and their processing (type of thermal processing, surface roughness) on the tribological and mechanical characteristics of the formed composites. These approaches help simplify the process and avoid lengthy experimental procedures [17].

Veeresha et al. investigated the influence of B_4_C micro-sized particle addition on the mechanical and tribological characteristics of the Al2618 base alloy [18]. Composites were produced via the stir casting route; the size of the composites’ reinforcement particles was 63 µm, and weight percentages varied from 2 to 8 wt.%. In the microstructure of the composites, there was a uniform distribution of B_4_C reinforcing particles in the Al2618 base, regardless of varying weight percentages. The microstructure showed the absence of gaps, voids, or cold closes, indicating strong interfacial bonding between the B_4_C particles and the Al2618 alloy base. Mechanical characteristics like hardness, ultimate tensile strength, yield strength, and compression strength increased with an increase in wt.% B_4_Cin composite, while elongation decreased as well as tribological characteristics like wear loss. Varol and Canakci [19] developed two novel milling models to investigate the influence of reinforcement particle size in Al2024-B_4_C composites. These composites, which incorporated B_4_C particles at varying weight percentages (0, 5, 10, and 20 wt.%), with two particle sizes (49 μm and 5 μm), were produced through mechanical milling. The significant impact of milling time, reinforcement size, and content on the resulting particle size and microhardness of the composite powders was observed. With an increase in milling time, the size of coarse B_4_C particles decreased from 122 to 27 nm, while the size of coarse B_4_C particles decreased to 18 nm. Microhardness increased with the milling time and weight percentage of B_4_C particles. Abdollahi et al. [4] investigated the mechanical and tribological characteristics of Al2024 alloy-based composites produced by mechanical milling and hot extrusion before and after adding 5 wt% B_4_C particles with a particle size, of 20 µm before milling. The crystallite size of the unreinforced alloy was reduced from 107 nm to 48 nm, while the Al2024-B_4_C nanocomposite crystallite size was reduced to 31 nm. After mechanical and wear tests, it was concluded that mechanical milling and the presence of B_4_C particles increased the strength and hardness of the Al2024 alloy but decreased its ductility and wear rate. Luo et al. [20] investigated the influence of B_4_C content on the microstructure and mechanical behavior of Al2024-B_4_C composites fabricated by plasma activated sintering. During the fabrication of composites for the Al alloy, reinforcing B_4_C particles with an average size of 4.2 µm and a weight percentage of 7.5, 17.5, or 27.5 wt.% were added. The observed mechanical characteristics, notably the Vickers hardness and yield strength of composite Al2024 + 17.5 wt.% B_4_C, were significantly improved due to the network reinforcement architecture. In the microstructure of the composite with the 27.5 wt.% B_4_C particles, there was severe aggregation of particles, visible cracks, and pores, which all led to a decrease in the relative density and mechanical characteristics when compared to the Al2024 + 17.5 wt.% B_4_C composite. Aluminum base micro and nanocomposites with B_4_Creinforcement particles fabricated ultrasonic cavitation-assisted stir casting were investigated by Harichandran and Selvakumar [21]. The amount of addition of both micro and nano B_4_C particles was from 2 to 10 wt.%. The hardness of composites with microsized reinforcement particles increased significantly with an increase in B_4_C addition up to 8 wt.%. The composite with nano B_4_C particles, had the same trend of increase in hardness, but it was not so significantly increased. With the addition of 10 wt.% of both micro and nano B_4_C particles hardness decreased. The lowest wear rate and coefficient of friction were observed for composites with 8 wt.% B_4_C nanoparticles, and it was concluded that the formation of a mechanically mixed layer is important in controlling the wear behavior of the Al-B_4_C micro and nanocomposites.

Optimization is very important in the modern industry in terms of cost and time reduction in product development. Prediction of the effect of reinforcement on the physical and mechanical properties of Al2024-B_4_C composites with the application of ANN was investigated by Varol et al. [22]. Composites were produced with powder metallurgy with different milling times (0–10 h) and different B_4_C weight percentages (5, 10, and 20 wt.%) and sizes (5 and 49 µm). It was concluded that with an increase in B_4_C wt.%, density decreases while hardness increases for both B_4_C particle sizes. The highest tensile strength was observed for composites with 10 wt.% B_4_C, a particle size of 5 µm, and a milling time of 5 h. The developed ANN model gave a good correlation between predicted and experimental results with a low error %. Canakci et al. [23] used ANN to predict the abrasive wear behavior of composites with AA2024 base alloy reinforced with B_4_C particles. Composites were produced via the stir casting method with different volume percentages and particle sizes of reinforcement. For the ANN training of the experimental data, there was one input layer (reinforcement particle size, sliding time, and volume percentage), two hidden layers with 6 and 4 neurons, respectively, and one output layer (volume loss, specific wear rate, and surface roughness). It was concluded that the addition of B_4_C particles to the base alloy improved the wear properties of the alloy and that ANN training gave a good correlation between experimental and predicted results. Another optimization method is the Taguchi method, which was applied by Soy et al. for the evaluation of the wear behavior of A360-based and SiC or B_4_C reinforced composites [24]. The amount of SiC and B_4_C reinforcement was 17 wt.%, and when compared to the base alloy, composites had a lower specific wear rate. After application of Taguchi method, it was observed that the highest influence on specific wear rate had type of material with 48.13%, followed by normal load with 31.83% and sliding speed with 8.77%. Using the optimal variant of parameters, the estimated S/N ratio for a specific wear rate was calculated, and a good correlation between predicted and actual values at a confidence level of 99.5% was observed. Verma et al. investigated the influence of load, sliding distance, and sliding speed on the tribological characteristics of an Al-B_4_C functionally graded composite using the Taguchi method. The composites were produced by a horizontal centrifugal casting process, studying the distribution of particles, density, porosity, and hardness in different zones. The research conclusions indicate a significant influence of the amount of B_4_C particles on the wear rate and friction coefficient, as well as on changes in wear behavior under different loads and sliding speeds. Also, they identified the optimal parameters that minimize the wear of the samples, and the examination of the microstructure revealed different wear mechanisms [25]. Kumar et al. investigated the tribological characteristics of self-lubricating Al–Mg–Si composites reinforced with MoS_2_–B_4_C particles. The content of B_4_C particles (x = 3.5, 7.0, 10.5, 14.0, and 17 wt%) was varied, while MoS_2_ was fixed at 3.0 wt%, forming composites primarily for use in automotive engine components. It was found that an improvement in wear resistance and coefficient of friction is achieved when MoS_2_ is incorporated into hybrid metal matrix composites (HMMCs), compared to only B_4_C reinforced composites [26]. A similar study was conducted where the influence of B_4_C (2.5, 5, and 10 vol.%) in Al7075 base was analyzed. The wear of the developed nanocomposites, produced by the powder metallurgy technique, was analyzed with the application of Taguchi L9 matrix, which consisted of the load, B_4_C content, speed, and sintering temperature. It was concluded that B_4_C is the most influential parameter and that with a higher reinforcement content in the base alloy, only abrasion occurs as the dominant wear mechanism, while with a lower reinforcement content, next to abrasion, delamination is also noted [27].

In recent studies, researchers have often chosen to use a combination of two or more optimization methods in order to verify the results before applying a certain optimization process in practice. This approach allows for more thorough and reliable verification, thereby reducing the risk of errors and improving the final efficiency of the process. The wear behavior of Al2618-B_4_C-Gr composites was analyzed with a combination of Taguchi and ANN methods [28]. Using the Taguchi method, it was determined that the load has the greatest influence on the composite’s wear, which was confirmed by ANN. In addition, it was noted that B_4_C and Gr particles were homogeneously distributed in the Al2618 base, and that particles created a dislocation barrier and contributed to the increase in hardness. It was concluded that the wear resistance is directly related to the hardness, and the addition of B_4_Cand Gr particles has the potential to increase the wear resistance of the base alloy. Another successful application of the Taguchi and ANN methods is in the optimization of squeeze casting process parameters for the AA6061-Al_2_O_3_-SiC-Gr composite. Using the Taguchi method, the optimal conditions for maximum hardness (131 HV) and tensile strength (329 MPa) were identified. ANN predicted hardness and tensile strength with 95% accuracy, showing higher accuracy compared to the regression model and experimental data [29].

According to the stated research and considering studies on Al-B_4_C composites in general, it is observed that the ceramic particle content in the aluminum alloy varies from 5 to 20 wt.%. Additionally, there is significant variation in the milling time during the composite production process, ranging from 5 to 20 h. Due to these variations; researchers have focused on the application of advanced software tools for optimization. The goal of optimization is to achieve composites with specific characteristics in the shortest possible time and with reduced production costs. The use of software tools provides efficient adjustment of process parameters, resulting in improved composite characteristics and more economical production processes.

In this study, hot pressed composites with AA2024 base alloy reinforced with B_4_C particles were investigated. This research uniquely combines hot pressing, setting it apart from previous studies that have primarily focused on conventional production methods. Through these innovative approaches, this study aims to achieve superior material properties. SEM microscopy was applied for the microstructural analysis of the produced composites. The aim of the present paper was to investigate the effect of B_4_C particle content, normal load, and milling time on the wear behavior of ceramic particle reinforced composites with the use of ANN and the Taguchi method. This study uniquely addresses the optimization of B_4_C particle content, specifically with the AA2024 base alloy and milling time, a combination less frequently investigated in existing literature. According to this, it fills a gap in current research, providing new insights into the microstructural and mechanical behavior of these composites.

## 2. Materials and Methodology

### 2.1. Procedure of Preparing Materials

In this study, AA2024 alloy powders were used as the base material for the production of A2024-B_4_C composites. These powders were obtained from Gündoğdu Exothermic Systems Casting Products Machinery Manufacturing Construction Industry and Trade Limited Co., İstanbul, Turkey. Sieving processes were initially applied to adjust the average particle size (d50) of the base AA2024 powders to 25 µm. The B_4_C powders used as the reinforcement material have a purity of 99.5% and an average particle size of 5 µm, and they were procured from Alfa-Aesar. AA2024 powders prepared by sieving were mixed with B_4_C particles at weight percentages of 0%, 5%, and 10%. These powder mixtures were subjected to mechanical milling in a Retsch PM200 planetary ball mill at a milling speed of 400 rpm. The milling durations were chosen as 0, 10, and 20 h. Higher B_4_C ratios and longer milling times can lead to issues such as sintering problems, porosity, and structural deformations in the final composite materials. Therefore, the selection of specific B_4_C ratios and milling times has been detailed in a previous study [30].

**Figure 1:**
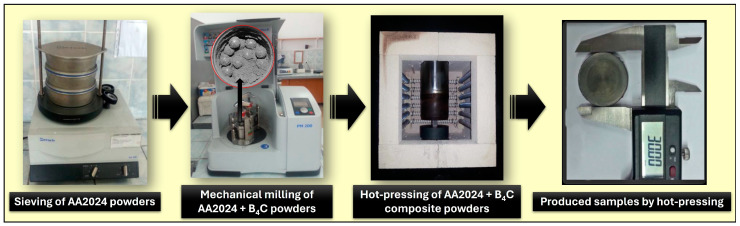
The production steps followed in the manufacturing of AA2024-B_4_C composites.

Tungsten carbide vials and balls were used in the milling process. The milling vial capacity is 125 mL, and the ball sizes are 5 mm. The ball-to-powder weight ratio used in the mechanical milling processes was selected as 10:1. Pre-shaping was first applied in a mold made of hot work tool steel to ensure better packing and sintering of the AA2024-B_4_C composite powders containing different ratios of B_4_C subjected to mechanical milling for different durations. The pre-shaping pressure was 600 MPa. The pre-shaped composite samples were held under vacuum at 560 °C for 3 h. At the end of this period, the samples were re-pressed at 600 MPa using a uniaxial press. More precisely, the samples were produced using the hot-pressing method. Cylindrical samples with a diameter of 30 mm were directly produced in molds with the relevant dimensions as wear test samples. The production steps followed in manufacturing AA2024-B_4_C composites are shown in Figure 1.

### 2.2. Design of Experiments and Experimental Results

After the preparation of sample materials, the experimental plan for tribological testing was developed. The selected parameters and their levels for experimental investigation are given in Table 1. All parameters are on three levels: more precisely, content of B_4_C (0, 5, and 10 wt.%), normal load (1, 5, and 10 N), and milingtime (0, 10, and 20 h). Based on the selected parameters and their levels, a Taguchi L27 matrix is developed for experimental investigation of the tribological behavior of aluminum composites. The 27 experiments were conducted according to Table 2, and each experiment was repeated five times.

The experimental findings have been converted into a signal-to-noise (S/N) ratio, with the signal representing the mean value and the noise indicating the standard deviation. Taguchi utilizes this ratio to evaluate quality characteristics across various types of loss functions. These relationships are distinguished into three types: “smaller is better”, “larger is better” and “nominal is best”. In this study, the emphasis is on minimizing, so the S/N ratio was determined using the provided equation. The S/N ratio for achieving the minimum WL (wear loss) and CoF (coefficient of friction) is expressed by the characteristic “smaller is better” and is calculated using the following equation [25,26]:

(1) $$S/N=-10log\frac{1}{n}\left(\sum y^{2}\right)$$

where: y—experimental results and n is the number of experiments. The obtained results of WL and CoF were converted into the S/N ratio.

### 2.3. Hardness and Wear Tests

The Brinell hardness of the hot-pressed samples was measured using a load of 32.5 kgf applied for 10 s. The mean hardness values were calculated from six indentations on each sample. The Brinell hardness test is often preferred for determining the hardness of micro-sized particle-reinforced aluminum matrix composites due to its suitability for rougher and less prepared surfaces. This is particularly important for aluminum matrix composites, where surface roughness and preparation can be significant factors. Additionally, the larger indentation left by the Brinell test averages out micro-level hardness variations, leading to more representative results. The Brinell method is also less sensitive compared to the Vickers and Rockwell methods, making it more suitable for materials like microparticle-reinforced aluminum matrix composites. Furthermore, the Brinell test’s ability to use larger loads provides a better evaluation of the material’s overall hardness behavior, which is beneficial for determining the macroscopic hardness of particle-reinforced composite materials. Therefore, despite the specific advantages of each method, the Brinell hardness test’s features make it a more suitable choice for these particular alloys [31,32,33].

Wear tests were conducted on samples produced by the hot-pressing method from AA2024-B_4_C composite powders, which were prepared with different B_4_C ratios and subjected to varying milling durations. The tests were carried out using a Ducom brand universal-type wear testing device. The type of wear test used was the ball-on-disc method. The wear tests were performed under loads of 1 N, 5 N, and 10 N at a sliding speed of 0.1 m/s, and each sample was tested for a sliding distance of 300 m. The wear loss was calculated by measuring the difference in the weights of the samples before and after the wear tests. During a wear test, the friction force values were obtained by determining the average values supported by the software. Since the literature emphasizes the effectiveness of ultrasonic cleaning in determining the true wear loss values, the test samples and abrasive ball surfaces were cleaned with an ultrasonic cleaner after each test.

### 2.4. Artificial Neural Networks

**Figure 2:**
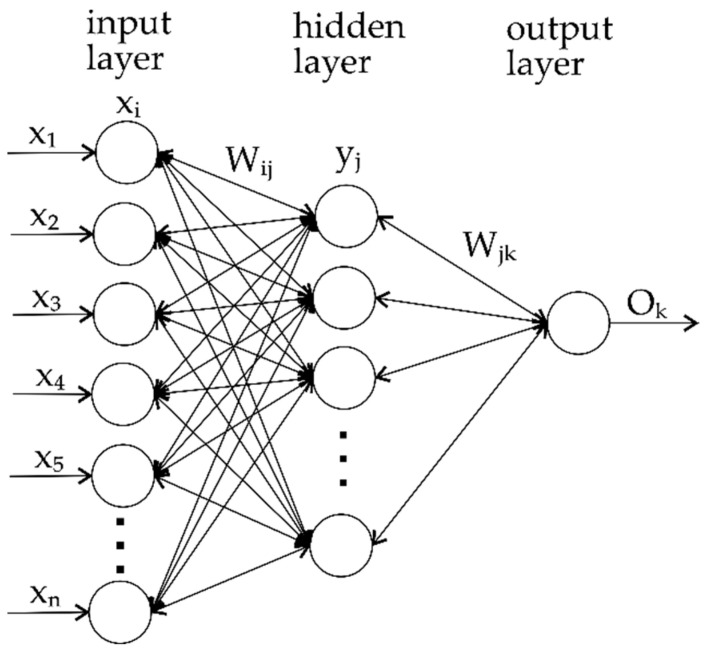
Schematics of basic ANN.

A type of machine learning that uses algorithms inspired by human brain functioning is artificial neural networks (ANNs). These networks are composed of interconnected nodes, often referred to as neurons, that are organized into layers. An ANN consists of an input layer, one or more hidden layers, and an output layer. The simplest ANN consists of an input layer, one hidden layer, and an output layer (Figure 2). Every layer has multiple neurons, and connections between neurons have associated weights, which determine the strength of the connection. These weights are adjusted based on training data during the learning process, allowing the network to learn relationships within the data.

For the development of ANN, the LM algorithm (TRAINLM) and feed-forward back propagation neural network are usually applied [17]. Transfer and activation functions are applied in order to approximate complex functions, thereby enhancing ANNs ability to solve a wide range of problems, from classification to regression. Activation functions that are usually applied in the training of ANN are hyperbolic tangent sigmoid (tansig) or log-sigmoid. Selecting the appropriate number of hidden layers in an ANN is challenging because it significantly impacts the model’s performance and computational efficiency. If too few layers are chosen, the model may suffer from underfitting, failing to capture the underlying patterns in the data. Conversely, too many layers can lead to overfitting, where the model learns the noise in the training data rather than the actual relationships, reducing its ability to generalize to new data. Both underfitting and overfitting can severely affect the model’s accuracy and increase its training time. Therefore, it is crucial to find a balance through careful experimentation and validation [34]. The number of neurons in the hidden layer and the transfer function are, usually, identified through an iterative trial-and-error process [35].

## 3. Results and Discussion

### 3.1. Taguchi Method

The Taguchi methodology is employed to efficiently determine optimal parameters by reducing the number of experiments by applying orthogonal arrays. In this experimental investigation, the focus is on achieving better performance by minimizing both WL and CoF.

After performing the experiments, S/N ratios for wear loss and CoF are were calculated for each parameter and for all levels (Table 3). Regardless of the applied quality characteristic, the results that are transformed into the S/N ratio are always interpreted in the same way more precisely, the higher the S/N ratio, the better.

**Figure 3:**
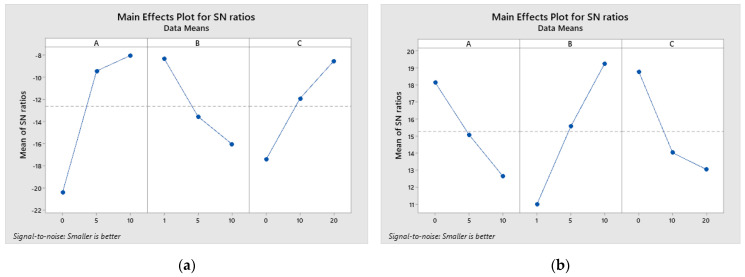
Main effects plot for S/N ratio for the (**a**) wear loss and (**b**) coefficient of friction.

Based on the calculated S/N ratios using the “smaller is better” characteristic, the results are shown in Table 3. The ranking of influential parameters in relation to WL and CoF is done by evaluating S/N ratios. Based on the delta value, the ranks of the considered parameters on WL and CoF are determined. Determining the optimal combination of parameter levels is possible based on Table 3 and the graphs shown in Figure 3.

According to the delta value, the rank of parameters based on the observed output tribological characteristics is determined. It is observed that the rank of parameters for WL is the content of B_4_C, followed by the parameter milling time, and the last is the normal load. While analyzing the results for CoF, it can be seen that the first ranking is normal load, the second is milling time, and the third is the content of B_4_C. The optimal parameter variant for WL and CoF is determined based on the graphs shown in Figure 3.

Based on the graphs (Figure 3), the optimal combination of parameter levels for wear loss was determined, which is A3, B1, and C3, while the optimal combination of parameters for CoF is A1, B3, and C1. Therefore, minimal wear loss of the composite is achieved with a reinforcement content of 10 wt.% B_4_C at a load of 1 N and a milling time of 20 h. The minimum CoF is achieved by a combination of a content of 0 wt.% B_4_C, more precisely without the content of the reinforcement, at a load of 10 N and a milling time of 0 h.

Research was conducted on experimental results to evaluate the influence of reinforcement content, normal load, and milling time on the WL and CoF of the composites. An analysis of variance (ANOVA) was performed at the 95% confidence level. The P value of the parameters indicates its influence, and if the P value is less than 0.05, it indicates that the parameter has a significant influence on the observed output (Table 4). Also, using ANOVA, the percentage influence of each parameter was calculated and is shown in the last column of Table 4.

Based on Table 4, it can be seen that WL is most affected by the content of B_4_C with 51.35%, followed by milling time with 22.75% and normal load with 17.54%. When the influence of observed parameters on CoF is analyzed, the normal load with an influence of 45.54% stands out, followed by milling time with 25.11% and the content of B_4_C with 20.22%. While considering the influence of the interaction of the parameters on the CoF, it can be stated that all the interactions of the considered parameters are significant according to the P-value, while it is observed that their percentage influence is less than 4%. Regarding the influence of the interaction of parameters on WL, the interaction of parameters A × B stands out with an influence of approximately 6%, while the influence of the interaction of parameters A × C and B × C is negligible.

After the analysis, the development of a mathematical model can be started in order to predict the wear and coefficient of friction for values that are not included in the experiment. Below are multiple linear regressions for wear loss and coefficient of friction:

WL = 10.32 − 3.453 × A + 3.18 × B − 0.978 × C + 0.2384 × A × A − 0.031 × B × B + 0.0209 × C × C − 0.2283 × A × B + 0.0998 × A × C − 0.0756 × B × C(2)

CoF = 0.1637 + 0.01436 × A − 0.03199 × B + 0.01953 × C − 0.000433 × A × A + 0.001827 × B × B − 0.000367 × C × C + 0.000184 × A × B − 0.000133 × A × C − 0.001011 × B × C(3)

Based on the results of this research, it was determined that the optimal content of B_4_C particles is 10 wt.% B_4_C, while in other studies, optimal values were 5 wt.% B_4_C [4,22], 8 wt.% B_4_C [18,21] and up to 20 wt.% B_4_C [19]. There are studies in which the characteristics of composites using a reinforcement content of up to 27.5% B_4_C were investigated, but it was concluded that the composite with 17.5% B_4_C showed the best tribological and mechanical characteristics [20]. Additionally, a good microstructure of the composite was observed by adding B_4_C particles [18,20], which affects the improvement of the mechanical and tribological characteristics of aluminum composites reinforced with B_4_C particles [4,18,19,21]. However, some researchers have concluded that excessive addition of these particles, such as 27.5 wt.% B_4_C, can negatively affect the properties of the composite [20]. Some recommendations for future research are that, next to the optimal reinforcement content of 10 wt.% in the base alloy, investigate composites with a higher B_4_C content. However, it is difficult to determine the exact percentage of the reinforcement content because there are many factors that affect the microstructure of the composite and its characteristics, such as the influence of the milling time, which can favorably affect the microhardness of the composite [19], as we have confirmed in this research.

### 3.2. ANN Model

**Figure 4:**
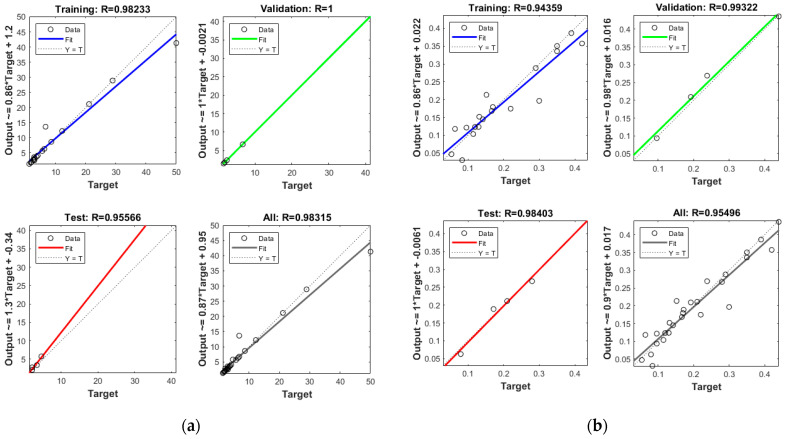
Regression coefficients for (**a**) wear loss and (**b**) CoF.

**Figure 5:**
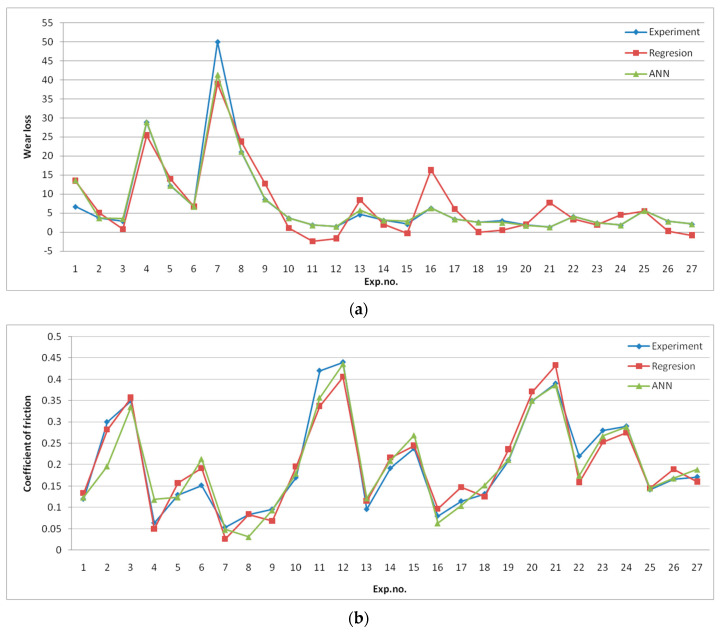
Comparative presentation of the results of the experiment, regression model, and ANN model for (**a**) wear loss and (**b**) CoF.

Before the selection of appropriate networks, the networks with different numbers in the hidden layer (5–30) and activation functions (tansig and logsig) were trained. In this paper, two feed-forward back propagation ANNs are used to predict wear loss and CoF separately. The selected input for both networks was the same (content of B_4_C, normal load, and milling time), as well as the number of neurons in the hidden layer (10 neurons) and activation function tansig, while only the outputs were different (wear loss and CoF). Based on the previously mentioned results, the best results were for architecture 3-10-1 of both networks. In Figure 4a,b, regression coefficients for training, testing, validation, and overall regression coefficients for trained networks are shown. It can be observed that the overall coefficient for wear loss was 0.98315 and the regression coefficient for CoF was 0.95496. The regression coefficient values higher than 0.95 suggest that there will not be large deviations from the experimental values with the ANN prediction, which can be proven with a comparison between the obtained results. The comparative view of experimental results, regression, and ANN prediction is given in Figure 5.

It can be observed that for wear loss, there is a good correlation between experimental and ANN results, while there is a greater deviation between experimental and regression results. Due to the complex nature of CoF, there is more deviation between experimental results, regression, and ANN predictions.

## 4. Microstructure and Worn Surfaces of Composites

**Figure 6:**
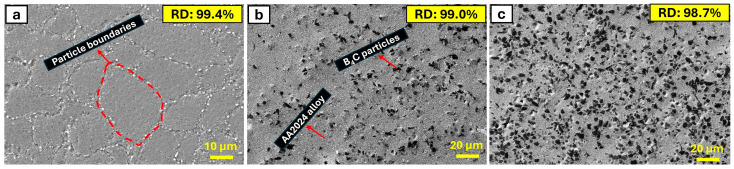
SEM images of the microstructure of (**a**) AA2024 alloy, (**b**) AA2024 + 5% B_4_C, and (**c**) AA2024 + 10% B_4_C composites with the RD values.

Figure 6 presents the internal structure of bulk samples produced by hot pressing AA2024 alloy and composites with 5 wt.% and 10 wt.% B_4_C obtained by hand-mixing the powders without milling. Figure 6a shows the internal structure of the unreinforced AA2024 base alloy, on which there are clearly noticeable boundaries between the particles. The internal structures of the composites with 5 wt.% and 10 wt.% B_4_C reinforcement are shown in Figure 6b and Figure 6c, respectively. The black regions observed in the internal structure of the composites represent the B_4_C particles. It is evident that the black regions in the internal structure obtained with 5 wt.% B4C reinforcement are significantly less than those obtained with 10 wt.% B_4_C reinforcement. Additionally, it is noteworthy that the particle boundaries observed in the internal structure of the unreinforced AA2024 begin to disappear with the addition of B_4_C. B_4_C particles enhance the diffusion of atoms within the AA2024 base during the sintering process [36]. The presence of these particles provides additional pathways or reduces the activation energy required for atomic movement, leading to a more uniform distribution of elements and the elimination of distinct particle boundaries. Adding B_4_C leads to solid solution strengthening, where the B_4_C particles dissolve partially in the aluminum base, altering its microstructure. This process disrupts the original grain boundaries, causing them to become less defined and more integrated into the overall base structure [37]. The presence of B_4_C particles acts as nucleation sites during the solidification and recrystallization processes, leading to finer grain sizes. Finer grains produce a more homogenous microstructure with less pronounced individual grain boundaries [38]. Furthermore, B_4_C particles impede the movement of dislocations within the base [36]. This pinning effect stabilizes the microstructure and prevents the growth of grains, resulting in a more uniform and less distinct grain boundary appearance. Additionally, it is well-known that an increase in the B_4_C reinforcement content leads to an increase in the porosity of the material’s microstructure because the increase in B_4_C reinforcement results from the hindering of hard particles during the sintering process of the composite powders. This situation inevitably leads to a decrease in the relative density (RD) values of the materials with an increase in the addition of hard particle additives, as seen in the RD values in Figure 6. While RD% values were determined by dividing the experimental density values by the theoretical density values, the porosity% values were calculated as in the previous study [30] by taking the difference of the obtained RD values from the 100% full density value. Accordingly, the calculated RD-porosity values for AA2024 alloy, AA2024 + 5% B_4_C, and AA2024 + 10% B_4_C composites are 99.4–0.6%, 99.0–1%, and 98.7–1.3%, respectively. The increase in porosity does not negatively affect the hardness and tribological properties of the material when the increase in porosity is low. This is because the B_4_C particles are exceptionally hard and wear-resistant, which compensates for the porosity. The inherent hardness and strength of B_4_C contribute to the overall hardness and wear resistance of the composite, counteracting any potential negative effects from increased porosity. Additionally, the distribution of B_4_C particles within the base can enhance load-bearing capacity and improve the overall tribological performance, thus maintaining or even enhancing the material’s hardness and wear properties despite the increased porosity. The porosity levels are low with the low content of B_4_C particles without heavy agglomeration in the microstructure [30].

**Figure 7:**
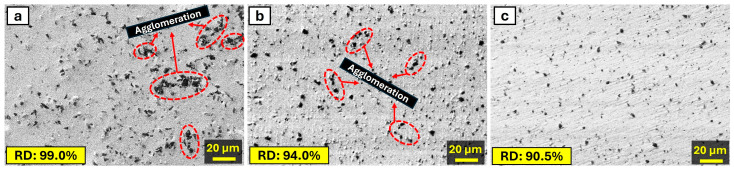
SEM images of the microstructure of AA2024 + 5% B_4_C obtained with (**a**) 0 h, (**b**) 10 h, and (**c**) 20 h milling with the RD values.

Figure 7 illustrates the change in the microstructure of composite samples obtained with a 5 wt.% addition of B_4_C to the AA2024 alloy with increasing milling time. Figure 7a describes the microstructure of samples produced by hot pressing after manually mixing composite powders without milling. Notably, in the composites produced without milling, the presence of B_4_C agglomeration regions is quite prominent. Increasing the milling time to 10 h significantly reduces the agglomeration regions compared to samples produced without milling (Figure 7b). A milling time of 20 h results in the homogeneous distribution of B_4_C particles in the microstructure, as evident in Figure 7c. The Taguchi method also showed that the milling time of 20 h was optimal for WL. The effect of milling time increases on the distribution of B_4_C particles in the microstructure can be explained by a reduction in agglomeration, enhanced mixing, increased surface area, particle size reduction, and the formation of new interfaces. The B_4_C particles may tend to agglomerate due to their high surface energy. Milling helps break down these agglomerates into smaller particles, promoting a more uniform dispersion throughout the base [39]. Longer milling times facilitate more thorough mixing of the B_4_C particles with the AA2024 alloy base. This leads to a more homogeneous distribution of the reinforcing phase within the composite structure [40]. As milling time increases, the surface area of the B_4_C particles also increases due to the mechanical milling action. This increased surface area promotes better bonding between the B_4_C particles and the base, improving the overall mechanical characteristics of the composite [41]. Prolonged milling reduces the size of B_4_C particles, making them more finely dispersed within the base. This finer dispersion enhances the strengthening effect of the B_4_C particles on the composite [42]. The mechanical action during milling creates fresh surfaces on both the B_4_C particles and the base material. This results in the formation of new interfaces, which can facilitate stronger bonding between the reinforcement and the base. This homogeneous distribution enhances wear resistance by preventing localized areas of weakness and promoting a more uniform load distribution during wear. The mechanical energy input during milling can cause the particles to harden. Work-hardened particles may not compact as efficiently during the hot pressing process, leading to an increase in porosity and a decrease in density. Increased milling time typically leads to a reduction in particle size. Smaller particles have a higher surface area-to-volume ratio, which can increase the likelihood of oxide formation and other surface contaminants that prevent complete densification during hot pressing. Due to these reasons, it is expected that the RD values decrease significantly from 99% to 90.5% with increasing milling time, as shown in Figure 7, and that the porosity values increase from 1% to approximately 10%. On the other hand, the extended milling time leads to a more homogeneous distribution of B_4_C particles within the AA2024 base. This enhanced dispersion ensures that the B_4_C particles are more evenly distributed throughout the composite, thereby providing better reinforcement and load-bearing capacity. Secondly, the increased milling time results in a refinement of the powder particles. Finer particles lead to a larger surface area for bonding during the hot pressing process, which can enhance the interfacial bonding between the matrix and the reinforcement particles. Stronger interfacial bonding contributes to the improved mechanical properties, such as increased hardness and strength. Finally, the refinement of the powder particles and the improved distribution of B_4_C also enhance the wear resistance of the composite. The well-dispersed and finely distributed B_4_C particles act as effective barriers against wear, improving the tribological properties of the composite [30].

### Investigation of Wear

**Figure 8:**
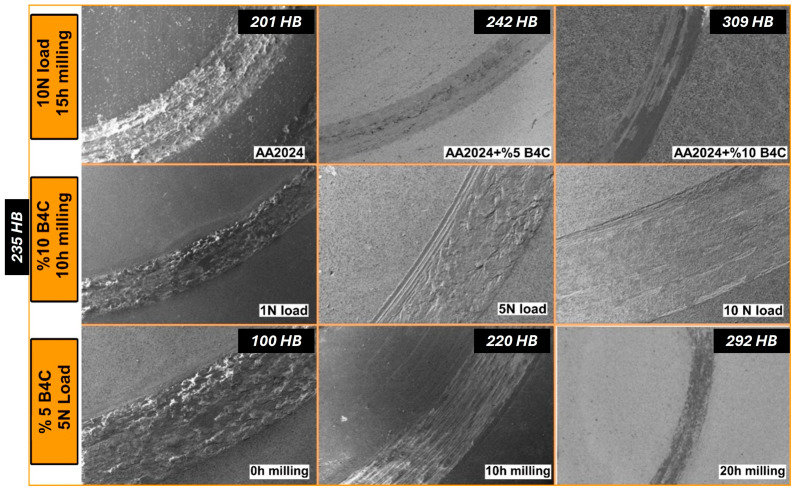
Wear tracks SEM images of the samples obtained under different parameters.

The most common method used to determine the wear phenomenon, specifically the wear losses examined in this study, is to analyze the weight losses and the sizes of wear scars formed during wear. A larger scar width indicates a higher wear loss, while a smaller one indicates a lower wear loss. Figure 8 shows the wear scars formed at different reinforcement contents, applied loads, and milling times. Initially, after examining the scars formed at constant load and milling time, it was observed that the scar widths decreased with the increasing amount of B_4_C. There are two main reasons for this: First, the B_4_C particles added to the structure increase the hardness of the composite, thus creating resistance to wear. As a result of this increased hardness, the amount of wear decreases, and consequently, the wear scar width diminishes. Additionally, the adhesion of B_4_C particles to the surface and the initial contact between the B_4_C particles and the counter surface significantly affect wear. B_4_C has a very high modulus of elasticity and shear resistance values. Therefore, during friction, the friction forces must exceed the shear resistance of B_4_C for wear to occur. However, in this study, the friction forces generated are insufficient to exceed the shear resistance of B_4_C. Thus, the continuous sliding motion impacts the interface bond formed between the B_4_C and the AA2024 base, causing the B_4_C particles to separate from the base phase. In other words, the counter surface must first overcome the B_4_C barrier to wear down the composite material. For all these reasons, as the amount of B_4_C increases, the amount of wear decreases, and the wear scar widths become smaller. Examining the SEM photographs obtained after wear tests performed under 1 N, 5 N, and 10 N normal loads on samples produced after 10 h of milling containing 10 wt.% B_4_C, it is observed that the smallest scar width occurs at 1 N load, and the largest scar width occurs at 10 N load. The increase in wear with the increasing load is an expected situation. Specifically, the separation and removal of B_4_C from the interface and achieving contact with the matrix in a shorter time are achieved more rapidly with this load increase. The increased load particularly causes more wear on the base material. When examining the scar widths varying with increasing milling time, it is seen that the scar widths decrease with increasing milling time. This situation is already clearly demonstrated in the weight loss measurements. The most important reason for the milling time’s effect on wear behavior is the increase in the hardness of the ground powders and, consequently, the hardness of the composites produced from these powders. In another study examining the properties of these powders and their mechanical characteristics, it was stated that the hardness of these powders increases with increasing milling time, and, accordingly, significant improvements in the hardness and strength values of the composites occur [30]. In terms of wear behavior, it can be stated that the increase in the hardness and strength values of the bulk sample due to the milling time directly leads to a decrease in the amount of wear and a reduction in the wear scar widths.B_4_C particles significantly enhance the hardness of the composites at consistent milling times. This improvement is attributed to the incorporation of reinforcement particles into the AA2024 base. Also, this increase is due to the relatively harder B_4_C particles embedded in the base, their exceptional resistance to indentation on the soft aluminum base, and the reduced particle size with further milling times. Additionally, the presence of B_4_C particles in the base and further milling times enhance strain hardening, further contributing to the increased hardness of the nanocomposites. Therefore, it is also evident from the worn surface images, which include hardness values in HB (Figure 8), that the increase in B_4_C content and milling time leads to an enhancement in the hardness of the composites. This increase in hardness is scientifically linked to the reduction in the wear scar width, consequently improving the wear resistance of the composites.

Additionally, the increase in the B_4_C ratio in the microstructure enhances the resistance of the AA2024 base material against abrasive wear due to its high hardness. Furthermore, an increase in milling time promotes the formation of uniformly distributed B_4_C within the microstructure, which minimizes wear by ensuring contact with hard B_4_C particles at each interaction with the abrasive ball. The most effective mechanism involves plastic deformation due to the presence of the soft AA2024 base, which initially causes surface scratching as the harder particles of B_4_C increase, leading to abrasive wear. The more contact with the abrasive occurs in softer areas, the greater the adhesion, whereas contact with particles by the abrasive results in either hard or soft particles causing scratching of the surface or delaminations demonstrating adhesive wear. Similar findings are supported by Ghassemi et al. in the literature [43]. Consequently, the amount and distribution of B_4_C in the microstructure directly affect wear resistance.

In this study, the optimization of the wear behavior of aluminum base composites reinforced with B_4_C was investigated. According to the literature, B_4_C increases the wear resistance of aluminum base composites due to their high hardness and thermal stability. B_4_C particles act as hard reinforcements that bear the load during the wear process, reducing the direct contact between the aluminum base and the wearing surface thus minimizing wear. Additionally, B_4_C’s thermal stability helps to maintain the integrity of the composite structure under high temperatures generated during wear [44,45,46,47,48]. Furthermore, increasing milling time enhances the homogeneity of particle distribution and refines the microstructural characteristics of the composites. Prolonged milling leads to better dispersion of B_4_C particles within the aluminum base, reducing agglomeration and promoting uniform load distribution. These homogeneous distributions are crucial for the improvement of mechanical characteristics and wear resistance as they minimize weak points within the composite. The refinement of the microstructure due to extended milling time also contributes to the improved wear behavior. Fine-grained structures have higher hardness and strength, which in turn increases resistance to wear. The refinement process involves the breaking down of larger grains into smaller ones, which enhances the overall hardness of the composite [49,50,51]. The theoretical test results of Taguchi and ANOVA, consistent with these theoretical foundations, indicate that both the increase in B_4_C reinforcement and extended milling time significantly improve wear properties. Specifically, the interaction between B_4_C particles and the aluminum base becomes more effective with prolonged milling, leading to a more resilient composite material. These findings are supported by existing theories in the literature [52,53,54,55] and provide important insights for the design and optimization of aluminum base composites, especially in applications requiring high wear resistance.

## 5. Conclusions

In summary, based on the design of the experiments, the dry sliding tribological investigation of AA2024 + B_4_C composites was performed in this study. The content of B_4_C reinforcement particles and milling time were varied during the production of composites. Based on the microstructural analysis of the composite, the addition of B_4_C particles to the AA2024 base improves the structure of the composite, making the particle boundaries less pronounced, resulting in a more homogeneous microstructure and smaller grain boundaries. Additionally, it was concluded that an increase in the milling time of composite powders with 5% B_4_C wt.% significantly reduces agglomeration and leads to a more homogeneous distribution of B_4_C particles in the AA2024 base.

The tribological experiments were done based on L27 base for different B_4_C contents (0, 5, 10 wt.%) under loads of 1, 5, and 10 N and for milling times of 0, 10, and 20 h. The aim was to investigate the influence of the B_4_C content, applied load, and milling time on the wear of the AA2024 composite. It was observed that an increase in B_4_C content and milling time reduces the width of the wear track due to the increased hardness of the composite, which was specified in another study [30].

In terms of reproducibility, the experiments were conducted under controlled conditions using standardized procedures to ensure consistency. The materials and methods, including the specific proportions of B_4_C and milling times, were meticulously documented to allow replication by other researchers. Multiple trials were performed to verify the consistency of the results, indicating that the observed improvements in wear resistance due to B_4_C reinforcement and milling time are reliable and reproducible. Regarding industrial applicability, the findings of this study hold significant potential for industries where wear resistance is crucial, such as automotive, aerospace, and manufacturing. The enhanced wear properties of aluminum base composites with B_4_C reinforcement can lead to a longer component lifespan and reduced maintenance costs. The integration of these composites into industrial processes can be facilitated by their compatibility with existing manufacturing techniques such as powder metallurgy and casting. Scalability is a key consideration for industrial applications. The milling process and B_4_C reinforcement used in this study can be scaled up for mass production. However, challenges such as ensuring uniform particle distribution and maintaining consistent mechanical characteristics across larger volumes need to be addressed. Economic factors, including the cost of B_4_C and the milling process, should also be considered. Future research could focus on optimizing these parameters to make the production process more cost-effective and efficient on an industrial scale.

Using the Taguchi method, the parameters affecting wear and the coefficient of friction of the AA2024 composite with B_4_C were identified and ranked. It was concluded that the B_4_C content primarily influences wear, whereas normal load is the key factor affecting CoF. Optimal combinations for minimizing wear include 10 wt.% B_4_C, 1 N load, and 20 h milling time, while for minimizing CoF, the optimal parameters are 0 wt.% B_4_C content, 10 N load, and 0 h milling time. ANOVA analysis confirmed the significant influence of these parameters on WL and CoF. The results of ANN prediction show that there is a good correlation between predicted and experimental values of wear loss and CoF. The application of ANNs for the optimization of wear loss and COF gives insights for future improvements in efficiency, cost reduction, and performance enhancement of various mechanical systems and components. With the use of ANN, researchers and engineers can unlock new insights and strategies for mitigating wear and friction in diverse applications.

The findings of this study have significant implications for the development and optimization of aluminum base composites in various industrial applications. The enhanced wear resistance and optimized frictional properties of the AA2024 + B_4_C composites suggest their potential use in high-stress environments such as automotive, aerospace, and manufacturing industries where material durability is critical. The ability to fine-tune the composite properties through controlled variations in B_4_C content and milling time provides a versatile approach to meeting specific performance requirements. The reduction in wear and friction achieved through the optimal combinations of reinforcement and process parameters can lead to longer-lasting components, reduced maintenance costs, and improved overall system reliability. These improvements are particularly relevant in applications where minimizing downtime and extending the service life of components are essential. Moreover, the successful application of the Taguchi method and ANN predictions demonstrates the potential of these advanced statistical and computational tools in materials engineering. They offer a systematic and efficient way to explore and optimize complex material systems, paving the way for more informed and data-driven decisions in composite design and processing. In conclusion, this study not only advances the understanding of the tribological behavior of AA2024 + B_4_C composites but also highlights the practical benefits and industrial relevance of these materials. The insights gained from this research can guide future studies and applications, contributing to the development of more durable and efficient materials for a wide range of technological applications.

## Figures and Tables

**Table 1:** Control parameters and their levels.

Input Parameters	Unit	Level 1	Level 2	Level 3
A: Content of B_4_C	wt.%	0	5	10
B: Normal load	N	1	5	10
C: Miling time	h	0	10	20

**Table 2:** Experimental design using L27 orthogonal array and experimental results.

Exp. No.	A	B	C	WL, mg	CoF	S/N for WL	S/N for CoF
1	0	1	0	6.7	0.12	−9.5424	18.4164
2	0	1	10	3.7	0.3	−5.1055	10.4576
3	0	1	20	2.9	0.35	−2.2789	9.1186
4	0	5	0	28.9	0.064	−12.2557	23.8764
5	0	5	10	12.2	0.13	−7.6042	17.7211
6	0	5	20	6.7	0.152	−5.1055	16.3631
7	0	10	0	50	0.054	−14.9638	25.3521
8	0	10	10	21.1	0.084	−8.9432	21.5144
9	0	10	20	8.6	0.096	−6.4444	20.3546
10	5	1	0	3.7	0.17	−9.5424	15.3910
11	5	1	10	1.9	0.42	−5.1055	7.5350
12	5	1	20	1.5	0.44	−2.2789	7.1309
13	5	5	0	4.6	0.096	−12.2557	20.3546
14	5	5	10	3.1	0.192	−7.6042	14.3340
15	5	5	20	2.1	0.238	−5.1055	12.4685
16	5	10	0	6.3	0.08	−14.9638	21.9382
17	5	10	10	3.4	0.115	−8.9432	18.7860
18	5	10	20	2.6	0.132	−6.4444	17.5885
19	10	1	0	3	0.21	−9.5424	13.5556
20	10	1	10	1.8	0.35	−5.1055	9.1186
21	10	1	20	1.3	0.39	−2.2789	8.1787
22	10	5	0	4.1	0.22	−12.2557	13.1515
23	10	5	10	2.4	0.28	−7.6042	11.0568
24	10	5	20	1.8	0.29	−5.1055	10.7520
25	10	10	0	5.6	0.142	−14.9638	16.9542
26	10	10	10	2.8	0.167	−8.9432	15.5457
27	10	10	20	2.1	0.172	−6.4444	15.2894

**Table 3:** Responses of S/N ratios for smaller is better characteristics.

	WL			CoF		
Level	A	B	C	A	B	C
1	−20.417	−8.280	−17.454	18.13	10.99	18.78
2	−9.434	−13.551	−11.918	15.06	15.56	14.01
3	−8.027	−16.047	−8.506	12.62	19.26	13.03
Delta	12.390	7.767	8.948	5.51	8.27	5.75
Rank	1	3	2	3	1	2

**Table 4:** Responses Tables for Signal to Noise Ratios Smaller is better.

**Wear Loss (R-Sq = 99.20%, R-Sq(adj) = 97.40%)**							
Source	DF	Seq SS	Adj SS	Adj MS	F	P	%
A	2	828.40	828.400	414.200	257.18	0.000	51.35
B	2	283.00	283.003	141.502	87.86	0.000	17.54
C	2	367.08	367.076	183.538	113.96	0.000	22.75
A × B	4	96.61	96.614	24.153	15.00	0.001	5.99
A × C	4	17.89	17.891	4.473	2.78	0.102	1.11
B × C	4	7.36	7.360	1.840	1.14	0.403	0.46
Residual Error	8	12.88	12.885	1.611			0.80
Total	26	1613.23					100.00
**CoF (R-Sq = 99.48%, R-Sq(adj) = 98.30%)**							
Source	DF	Seq SS	Adj SS	Adj MS	F	P	%
A	2	137.126	137.126	68.563	319.72	0.000	20.22
B	2	308.856	308.856	154.428	720.12	0.000	45.54
C	2	170.285	170.285	85.142	397.03	0.000	25.11
A × B	4	26.959	26.959	6.740	31.43	0.000	3.98
A × C	4	17.412	17.412	4.353	20.30	0.000	2.57
B × C	4	15.811	15.811	3.953	18.43	0.000	2.33
Residual Error	8	1.716	1.716	0.214			0.25
Total	26	678.164					100.00

DF—degree of freedom, Seq SS—Sequential sum of squares, Adj SS—Adjusted sum of squares, Adj MS—Adjusted mean square.

## Data Availability

The raw data supporting the conclusions of this article will be made available by the authors on request.
